# Torulaspora lindneri sp. nov., a novel ascomycetous yeast species isolated from China and Thailand

**DOI:** 10.1099/ijsem.0.006789

**Published:** 2025-05-16

**Authors:** Shuang Hu, Sasitorn Jindamorakot, Qi-Yang Zhu, Liang-Chen Guo, Somjit Am-In, Jutaporn Sawaengkaew, Fitsum Tigu, Pei-Jie Han, Feng-Yan Bai

**Affiliations:** 1 State Key Laboratory of Mycology, Institute of Microbiology, Chinese Academy of Sciences, Beijing 100101, PR China; 2 College of Life Sciences, University of Chinese Academy of Sciences, Beijing 100049, PR China; 3 Microbial Diversity and Utilization Research Team, Thailand Bioresource Research Center, National Center for Genetic Engineering and Biotechnology, National Science and Technology Development Agency, Khlong Luang, Pathum Thani 12120, Thailand; 4 Department of Microbiology, Faculty of Science, Khon Khen University, Khon Khen 40002, Khon Khen, Thailand; 5 Department of Microbial, Cellular and Molecular Biology, Addis Ababa University, Addis Ababa 1176, Ethiopia

**Keywords:** Ascomycetous yeast, phylogeny, taxonomy, *Torulaspora lindneri *sp. nov.

## Abstract

A yeast strain isolated from chestnut tree bark collected in Guangshan County, Xinyang, Henan Province, China and a strain isolated from soil collected in Phu Wiang District, Khon Khen Province, Thailand possess identical sequences in the 26S ribosomal RNA gene D1/D2 domain and the internal transcribed spacer (ITS) region. Phylogenetic analysis showed that the two strains belong to the genus *Torulaspora* and are closely related to *T. jiuxiensis* but differed from the species by 7 (1.26%, 7 substitutions and 0 gap) and 43 (5.75 %, 31 substitutions and 12 gaps) mismatches in the D1/D2 domain and ITS region, respectively. The result suggests that the Chinese and the Thai strains represent a novel species in the genus *Torulaspora*, for which the name *Torulaspora lindneri* sp. nov. (holotype strain CGMCC 2.7783) is proposed. This species forms one or two spherical ascospores in persistent asci, usually with tapered protuberances.

## Introduction

The genus *Torulaspora* belonging to *Saccharomycetes*, *Saccharomycetales*, *Saccharomycetaceae* was first established by Lindner in 1904 to accommodate *Torulaspora delbrueckii* [1,2]. However, Lodder and Kreger van Rij merged *Torulaspora* and *Zygosaccharomyces* into the genus *Saccharomyces*, and then van der Walt and Johanssen reinstated the genera *Torulaspora* and *Zygosaccharomyces* in 1975 [3,4]. So far, the genus *Torulaspora* contains a total of ten species, namely *T. delbrueckii*, *T. franciscae* (isolated from soil in Spain), *T. globosa* (isolated from soil in the West Indies), *T. maleeae* (isolated from leaf of *Rhizophora stylosa* in Japan) [5], *T. microellipsoides* (isolated from apple juice in Germany), *T. pretoriensis* (isolated from soil in South Africa), *T. quercuum* (isolated from tree of *Quercus* sp. in China) [6], *T. indica* (isolated from soil in India) [7], *T. nypae* (isolated from nipa inflorescence sap in Thailand) [8] and *T. jiuxiensis* (isolated from rotting wood in China) [9]. The most typical feature of the species in this genus is that their asci with one to four spherical ascospores often have a small, tapered protuberance. Other properties of this genus include asexual reproduction occurring via multilateral budding on a narrow base, glucose and often other sugars are fermented and coenzyme Q-6 is produced, while the formation of true hyphae, nitrate assimilation and diazonium blue B tests are negative [4].

The *Torulaspora* species are widely distributed and exist in various habitats, including man-made and wild environments. * T. delbrueckii*, an ubiquitous yeast species, has been found in wine [10], beer [11], cider [12], brandy [13], apricot juice [14], cheese [15] and cocoa beans [16]. *T. globosa* has been found in lettuce rhizosphere [17] and rice/sugar cane leaves [18]. *T. pretoriensis* has been found in soil, cocoa fermentations and phylloplane [19]. In addition to their varied isolation substrates, certain species of the genus *Torulaspora* exhibit a broad geographical distribution. For instance, *T. delbrueckii* has been reported in 37 countries across Asia, Europe, Africa, America and Oceania [20], while *T. globosa* has been identified in Asia, Africa, South America and Oceania [9]. In recent years, this genus has received widespread attention due to its value in various aspects such as biosynthesis, biotransformation, biocontrol and food fermentation. Therefore, it is necessary to explore their diversity, collect strain resources and conduct systematic classification research.

*Fagaceae* plants are considered important ecological niches for yeasts (especially *Saccharomyces* species) in nature [21]. During an investigation of yeast diversity in chestnut (*Castanea mollissima*) forest from Xinyang, Henan Province, 44 isolates in the samples were identified as 17 known species: *Saccharomyces cerevisiae*, *Lachancea fermentati*, *Lachancea thermotolerans*, *Nakaseomyces glabratus*, *Wickerhamomyces anomalus*, *Meyerozyma caribbica*, *Millerozyma farinose*, *Yarrowia lipolytica*, *Curvibasidium nothofagi*, *Candida melibiosica*, *Hyphopichia burtonii*, *Maudiozyma exigua*, *Pichia kudriavzevii*, *Pichia manshurica*, *Schizosaccharomyces japonicus*, *Schwanniomyces polymorphus* and *Torulaspora pretoriensis*. A new ascomycetous yeast species belonging to the genus *Torulaspora* was discovered. Another strain belonging to the same new species was isolated from soil collected in Phu Wiang District, Khon Khen Province, Thailand. The new species is described based on detailed phylogenetic and phenotypic characterization in this study.

## Yeast isolation and phenotypic characterization

A total of 25 bark samples of chestnut trees were collected using 50 ml sterile centrifuge tubes from Guangshan County, Xinyang, Henan, China (114.9188° E, 32.0099° N) in August 2023 and transferred to the laboratory at room temperature. Approximately 30 ml of yeast extract peptone dextrose (YPD) broth (w/v, 1% yeast extract, 2% glucose and 2% peptone) supplemented with 7% ethanol and 200 µg ml^−1^ chloramphenicol was added to every 50 ml sterile centrifuge tube containing a bark sample and was incubated at room temperature for 3–7 days for the enrichment of yeasts in the sample. When bubbles or yeast cell precipitates were generated in the tube, the enrichment culture was shaken and diluted to a suitable gradient, and then an aliquot of 200 ml of the dilution was spread on a YPD agar plate supplemented with 200 µg ml^−1^ chloramphenicol. After 3–5 days of incubation at 25 °C, yeast colonies with different appearances were picked. The strains were then purified and preserved in 20% glycerol at −80 °C. The strain KKU-PM21 was isolated from a soil sample collected from Phu Wiang District, Khon Khen Province, Thailand (102.5833° E, 16.5000° N) in January 2011. Isolation of the strain KKU-PM21 was carried out by the enrichment technique using yeast extract-malt extract (YM) broth (w/v, 0.3% yeast extract, 0.3% malt extract, 0.5% peptone, 1% glucose) supplemented with 0.2% sodium propionate and 100 µg ml^−1^ chloramphenicol. After incubating on a rotary shaker at 120 r.p.m. at 25 °C for 2 days, the culture broth was spread on YM agar supplemented with 0.2% sodium propionate and 100 µg ml^−1^ chloramphenicol and incubated at 25 °C for 2–3 days; then, the yeast strain was purified on YM agar and preserved in YM supplemented with 10% glycerol at −80 °C.

Morphological, biochemical and physiological characterizations were performed according to the methods described by Kurtzman [4]. Assimilation of carbon and nitrogen compounds was examined in liquid media. Fermentation tests were done with inverted Durham tubes. The formation of vegetative cells was investigated on YM agar at 25 °C for 3 days. The formation of sexual structures was investigated on corn meal agar (2.5% corn starch, 2% agar), malt extract agar (5% malt extract, 2% agar), potato dextrose agar (20% potato infusion, 2% glucose, 1.8% agar), V8 agar (10 % V8 juice, 2% agar) and yeast carbon base agar (1.17% yeast carbon base, 2% agar) at 25 °C for 4 weeks.

## Molecular phylogenetic analyses

Genomic DNA was extracted by using the method described by Wang and Bai [22]. The internal transcribed spacer (ITS) region and the D1/D2 domains of the LSU rRNA gene were amplified using primer pairs ITS1 and ITS4, and NL1 and NL4 [23,24], respectively. The purified amplicons were sequenced using the same primer pairs, and the nucleotide sequences were compared by using the BLAST search through GenBank. Sequence alignment was performed using MAFFT v. 7 and manually improved where necessary using mega v. 7 [25,26]. Phylogenetic analyses using the neighbour-joining (NJ) methods with Kimura’s two-parameter model were performed by mega v. 7 software based on the concatenated sequences of the ITS region and the D1/D2 domain [27]. The confidence levels of the clades were estimated through 1,000 replicates bootstrap analysis.

**Fig. 1.**
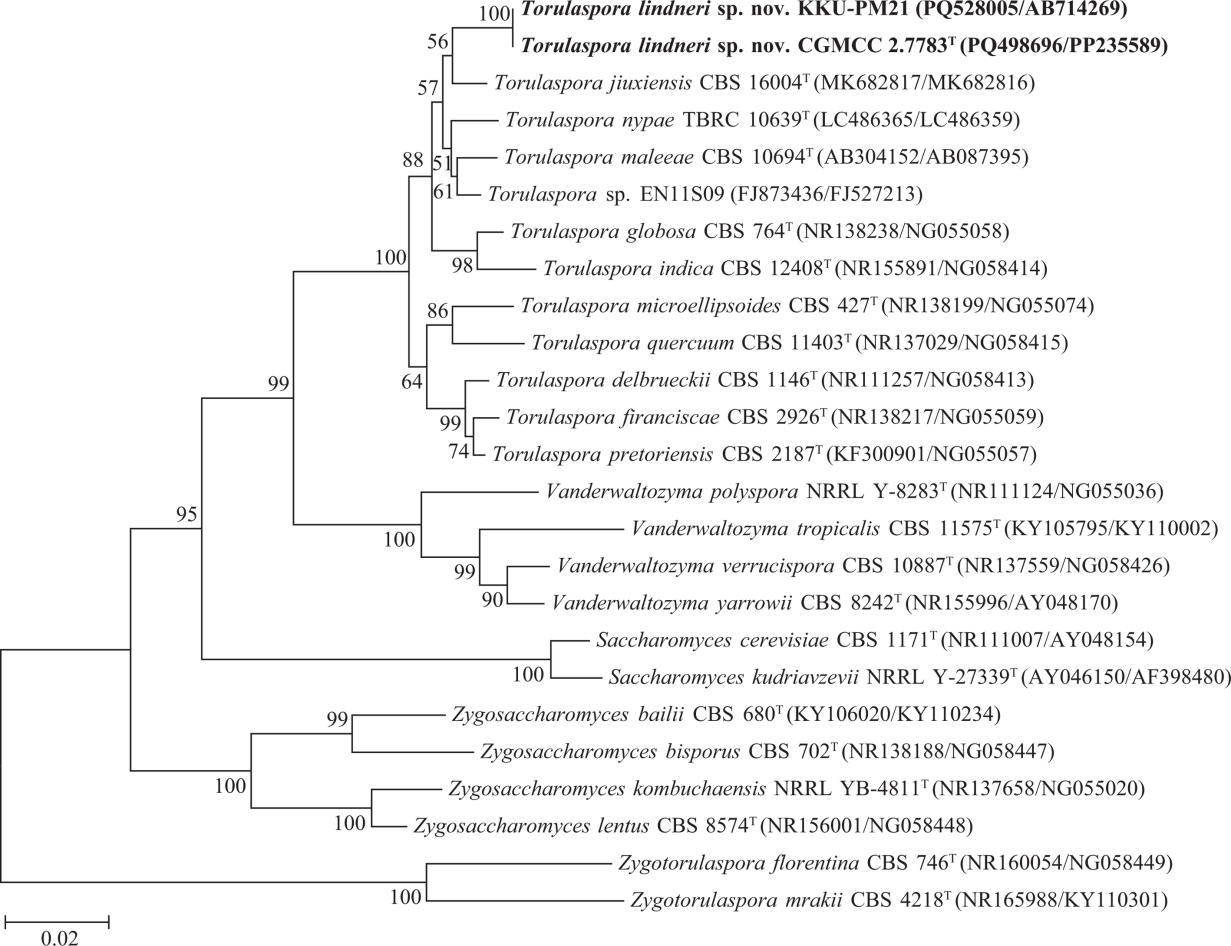
NJ phylogenetic tree showing the phylogenetic position of *Torulaspora lindneri* sp. nov. based on the D1/D2 domain and ITS region sequences. The two *Zygotorulaspora* species are used as the outgroup. Bootstrap values above 50% are shown on the branches. Type strains are denoted with the superscript ‘T’. Bar represents 0.02 substitutions per nucleotide position.

The sequence blast result indicated that strain CGMCC 2.7783 (original number HE144P-2) possessed identical D1/D2 and ITS sequences with strain KKU-PM21 (TBRC 19377) previously isolated from Thailand, suggesting that they are conspecific. The phylogenetic analysis based on the combined ITS and D1/D2 sequences showed that strains CGMCC 2.7783 and KKU-PM21 belong to the genus *Torulaspora* and are closely related to *T. jiuxiensis* (Fig. 1). Strain CGMCC 2.7783 differs from *T. jiuxiensis* CBS 16004^T^ by 7 (1.26%, 7 substitutions and 0 gap) and 43 (5.75 %, 31 substitutions and 12 gaps) mismatches, from *T. nypae* TBRC 10639^T^ by 9 (1.63%, 9 substitutions and 0 gap) and 46 (5.94 %, 42 substitutions and 4 gaps) mismatches, from *T. maleeae* CBS 10694^T^ by 9 (1.64%, 9 substitutions and 0 gap) and 51 (6.88 %, 49 substitutions and 2 gaps) mismatches, and from *Torulaspora* sp*.* EN11S09 by 4 (0.73%, 4 substitutions and 0 gap) and 39 (5.49 %, 35 substitutions and 4 gaps) mismatches, in the D1/D2 domain and ITS region, respectively. These results suggest that strains CGMCC 2.7783 and KKU-PM21 represent a novel species, for which the name *Torulaspora lindneri* sp. nov. is proposed.

## Phenotypical characteristics and ecology

In general, *T. lindneri* sp. nov. showed phenotypic characteristics typical of the genus *Torulaspora* but also distinguishable from its closely related species (Table S1, available in the online Supplementary Material). *T. lindneri* and the closest species *T. jiuxiensis* differ in the assimilation reactions of d-galactose, maltose, trehalose, melibiose, melezitose, d-xylose, ethanol, glycerol, ribitol, galactitol, d-mannitol, d-glucitol, dl-lactic acid, succinic acid, sodium citrate dihydrate and xylitol; the fermentation reactions of d-galactose, maltose, raffinose; and the growth ability at 37 ℃ and in 10% NaCl plus 5% glucose medium. However, *T. lindneri* and *T. maleeae* only differ in the fermentation reactions of raffinose, the assimilation reactions of α-Methyl-d-glucoside and the growth ability in 10% NaCl plus 5% glucose medium. *T. lindneri* and *T. nypae* differ in the fermentation reactions of maltose and lactose; the assimilation reactions of maltose, trehalose, melezitose, d-xylose, ethanol, glycerol, d-mannitol, d-glucitol, α-Methyl-d-glucoside, d-glucuronic acid, dl-lactic acid and xylitol; and the growth ability at 37 ℃ and on 60% glucose. Cell division occurs through multilateral budding from a narrow base, resulting in spherical to ellipsoidal budded cells. Cells frequently contain tapered protrusions reminiscent of conjugation tubes. The new species can strongly assimilate only limited carbon sources, including d-glucose, sucrose, maltose, melezitose, inulin and α-methyl-d-glucoside. *T. lindneri* sp. nov. can grow on 60% (w/v) glucose, suggesting its tolerance to high osmotic pressure. Two strains of *T. lindneri* were isolated from bark and soil in forest environment within tropical or subtropical monsoon climate zones, indicating that the species may have a close relationship with plants. Their colonization mechanisms and biological functions in different living environments need further exploration.

## Description of *Torulaspora lindneri* S. Hu and F.Y. Bai sp. nov

*Torulaspora lindneri* (lind’ ne.ri. N.L. gen. n. *lindneri,* of Paul Lindner, named in honour of his contribution to the establishment of the genus).

**Fig. 2.**
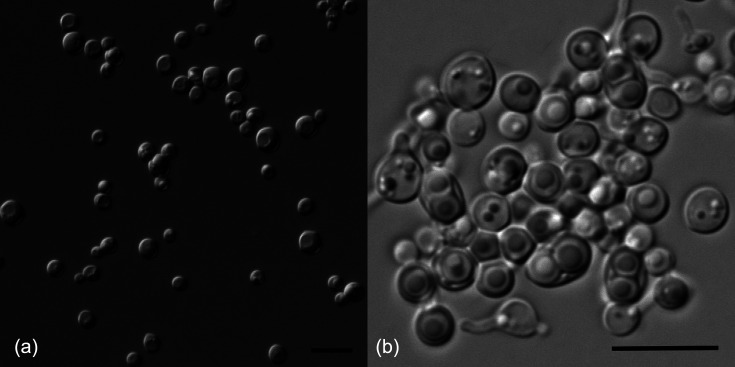
*Torulaspora lindneri* strain CGMCC 2.7783^T^. a: Vegetative cells on YM agar at 25 °C after 3 days. b: Asci with spherical ascospores and tapered protuberances on V8 agar at 25 °C after 15 days. Bars: 10 µm.

Culture characteristics: After the growth on YM agar for 3 days at 25 °C, colonies are white, butyrous, circular, slightly raised, with a smooth surface and entire margins. Cells are circular with a diameter of 2.5–5 µm. Budding is multilateral and pseudohyphae or true hyphae are not formed (Fig. 2a). After 1 month in YM broth at 25 °C, sediment is present, but no pellicle is observed. After the growth on V8 agar for 15 days at 25 °C, persistent asci are formed through conjugation between a cell and its bud or between independent cells, each containing one or two spherical ascospores (Fig. 2b).

Physiological and biochemical characteristics: Sugar fermentation: d-glucose (or weak), sucrose and maltose are positive, while raffinose, d-galactose, lactose and trehalose are negative. Carbon compound assimilation: d-glucose, sucrose (slow), maltose (slow), trehalose (weak), melezitose (or slow), inulin (or delayed) and α-methyl-d-glucoside (weak or slow) are positive, while d-galactose, l-sorbose, cellobiose, lactose, melibiose, raffinose (various), soluble starch, d-xylose, l-arabinose, d-arabinose, d-ribose, l-rhamnose, d-glucosamine, methanol, ethanol (or weak), glycerol, erythritol, ribitol, galactitol, d-mannitol (or weak), d-glucitol, salicin, d-glucuronic acid, d-galacturonic acid, dl-lactic acid, succinic acid, sodium citrate dihydrate, inositol, hexadecane, N-acetyl-d-glucosamine and xylitol are negative. Nitrogen compound assimilation: l-lysine (or weak) and ammonium sulphate are positive, while cadaverine dihydrochloride (or weak), potassium nitrate, ethylamine hydrochloride and sodium nitrite are negative. Growth in vitamin-free medium, 10% NaCl plus 5% glucose medium (weak) and on 50% and 60% (w/v) glucose are positive. Diazonium Blue B reaction, urease activity, liquefaction of gelatin and production of extracellular starch-like compounds are negative. The growth on YPD agar at 30 °C and 37 °C (weak) is positive, and negative at 40 °C.

The holotype CGMCC 2.7783 (original number HE144P-2) was isolated from the bark of a chestnut tree collected from Guangshan County, Xinyang, Henan, China, in August 2023, and was deposited in a metabolically inactive state in the China General Microbiological Culture Collection Centre (CGMCC), Beijing, China. An ex-type culture has been deposited in the Japan Collection of Microorganisms (JCM), Koyadai, Japan, as JCM 36914. The GenBank/EMBL/DDBJ accession numbers for the sequences of the D1/D2 domain of strains CGMCC 2.7783 and KKU-PM21 are PP235589 and AB714269, respectively, and of the ITS region are PQ498696 and PQ528005, respectively. The Fungal Names number is FN 572228.

## Supplementary material

10.1099/ijsem.0.006789 Uncited Table S1.

## Abbreviations

CGMCC: China General Microbiological Culture Collection Centre
ITS: internal transcribed spacer
JCM: Japan Collection of Microorganisms
NJ: neighbour-joining
YM: yeast extract-malt extract
YPD: yeast extract peptone dextrose

## References

[1] Groenewald M, Hittinger CT, Bensch K, Opulente DA, Shen XX (2023). A genome-informed higher rank classification of the biotechnologically important fungal subphylum *Saccharomycotina*. Stud Mycol.

[2] Lindner P (1904). Neue erfahrungen aus dem letzten jahre in bezug der hefen und Gärung. Jahrb Vers Lehranst Brau Berlin.

[3] Lodder J, Rij Nk (1952). The Yeasts, a Taxonomic Study, 1st ed.

[4] Kurtzman CP, Kurtzman CP, Fell JW, Boekhout T (2011). The Yeasts, a Taxonomic Study.

[5] Limtong S, Imanishi Y, Jindamorakot S, Ninomiya S, Yongmanitchai W (2008). *Torulaspora maleeae* sp. nov., a novel ascomycetous yeast species from Japan and Thailand. FEMS Yeast Res.

[6] Wang QM, Xu J, Wang H, Li J, Bai FY (2009). *Torulaspora quercuum* sp. nov. and *candida pseudohumilis* sp. nov., novel yeasts from human and forest habitats. FEMS Yeast Res.

[7] Saluja P, Yelchuri RK, Sohal SK, Bhagat G, Paramjit (2012). *Torulaspora indica* a novel yeast species isolated from coal mine soils. Antonie Van Leeuwenhoek.

[8] Kaewwichian R, Khunnamwong P, Am-In S, Jindamorakot S, Limtong S (2020). *Torulaspora nypae* sp. nov., a novel yeast species isolated from nipa (*Nypa fruticans* Wurmb.) inflorescence sap in southern Thailand. Int J Syst Evol Microbiol.

[9] Chu SB, Hu WT, Hui FL (2022). *Torulaspora jiuxiensis* sp. nov., a novel yeast species isolated from rotting wood. Int J Syst Evol Microbiol.

[10] Cheng Y, Geng S, Zhang J, Zhao X, Jiang J (2025). A comprehensive study on fermentation and aroma contributions of *Torulaspora delbrueckii* in diverse wine varieties: Insights from pure and co-fermentation studies. Food Res Int.

[11] Kayadelen F, Agirman B, Jolly NP, Erten H (2023). The influence of *Torulaspora delbrueckii* on beer fermentation. FEMS Yeast Res.

[12] Tocci N, Egger M, Hoellrigl P, Sanoll C, Beisert B (2023). *Torulaspora delbrueckii* strain behaviour within different refermentation strategies for sparkling cider production. Appl Sci.

[13] Liu J, Wan Y, Chen Y, Fan H, Li M (2024). Evaluation of the effect of *Torulaspora delbrueckii* on important volatile compounds in navel orange original brandy using E–nose combined with HS–SPME–GC–MS. Food Chem.

[14] Papun B, Wongputtisin P, Kanpiengjai A, Pisithkul T, Manochai P (2024). Fermentative characteristics and metabolic profiles of Japanese apricot juice fermented with *Lactobacillus acidophilus* and *Torulaspora delbrueckii*. Foods.

[15] Andrade GC, Andrade RP, Oliveira DR, Quintanilha MF, Martins FS (2021). *Kluyveromyces lactis* and *Torulaspora delbrueckii:* probiotic characterization, anti-salmonella effect, and impact on cheese quality. *LWT*.

[16] Visintin S, Ramos L, Batista N, Dolci P, Schwan F (2017). Impact of *Saccharomyces cerevisiae* and *Torulaspora delbrueckii* starter cultures on cocoa beans fermentation. Int J Food Microbiol.

[17] Cabrini PG, Sala FC, Magri MMR (2019). *Torulaspora globosa*: rhizosphere yeast promoting lettuce growth on seedlings and under field conditions. Hortic Bras.

[18] Nutaratat P, Srisuk N, Arunrattiyakorn P, Limtong S (2014). Plant growth-promoting traits of epiphytic and endophytic yeasts isolated from rice and sugar cane leaves in Thailand. Fungal Biol.

[19] Limtong S, Koowadjanakul N (2012). Yeasts from phylloplane and their capability to produce indole-3-acetic acid. World J Microbiol Biotechnol.

[20] Fernandes T, Silva-Sousa F, Pereira F, Rito T, Soares P (2021). Biotechnological Importance of *Torulaspora delbrueckii*: from the obscurity to the spotlight. *JoF*.

[21] Bai FY, Han DY, Duan SF, Wang QM (2022). The ecology and evolution of the baker’s yeast *Saccharomyces cerevisiae*. Genes.

[22] Wang QM, Bai FY (2008). Molecular phylogeny of basidiomycetous yeasts in the *Cryptococcus luteolus* lineage (*Tremellales*) based on nuclear rRNA and mitochondrial cytochrome b gene sequence analyses: proposal of *Derxomyces gen*. nov. and *Hannaella gen*. nov., and description of eight novel *Derxomyces* species. *FEMS Yeast Res*.

[23] Schoch CL, Seifert KA, Huhndorf S, Robert V, Spouge JL (2012). Nuclear ribosomal internal transcribed spacer (ITS) region as a universal DNA barcode marker for Fungi. Proc Natl Acad Sci USA.

[24] Kurtzman C, Robnett C (1998). Identification and phylogeny of ascomycetous yeasts from analysis of nuclear large subunit (26S) ribosomal DNA partial sequences. Antonie Van Leeuwenhoek.

[25] Katoh K, Standley DM (2013). MAFFT multiple sequence alignment software version 7: improvements in performance and usability. Mol Biol Evol.

[26] Kumar S, Stecher G, Tamura K (2016). MEGA7: molecular evolutionary genetics analysis version 7.0 for bigger datasets. Mol Biol Evol.

[27] Lachance MA (2022). Phylogenies in yeast species descriptions: in defense of neighbor-joining. Yeast.

